# Individually tailored nutritional guidance improved dietary intake of older family caregivers: a randomized controlled trial

**DOI:** 10.1007/s00394-022-02908-w

**Published:** 2022-05-27

**Authors:** Sohvi Koponen, Irma Nykänen, Roosa-Maria Savela, Tarja Välimäki, Anna Liisa Suominen, Ursula Schwab

**Affiliations:** 1 Institute of Public Health and Clinical Nutrition, School of Medicine, University of Eastern Finland, P.O. Box 1627, 70211 Kuopio, Finland; 2 Department of Nursing Science, University of Eastern Finland, P.O. Box 1627, 70211 Kuopio, Finland; 3 Institute of Dentistry, School of Medicine, University of Eastern Finland, P.O. Box 1627, 70211 Kuopio, Finland; 4 Department of Oral and Maxillofacial Diseases, Kuopio University Hospital, P.O. Box 100, 70029 KYS, Finland; 5 Department of Medicine, Endocrinology and Clinical Nutrition, Kuopio University Hospital, P.O. Box 100, 70029 KYS, Finland

**Keywords:** Dietary intake, Family caregiver, Nutritional guidance, Nutritional status, Older people

## Abstract

**Purpose:**

Older family caregivers (FCs) are vulnerable to insufficient dietary intake and risk of malnutrition. The aim of this study was to assess the impact of individually tailored nutritional guidance on the dietary intake and nutritional status of older FCs and their care recipients’ (CRs’) nutritional status.

**Methods:**

This study was a randomized controlled 6-month nutrition intervention in Eastern Finland. The inclusion criteria for FCs were having a home-living CR aged 65 or above and a valid care allowance. The exclusion criterion was CR receiving end-of-life care at baseline. Participants were randomly assigned to an intervention (FCs *n* = 63, CRs *n* = 59) and a control (FCs *n* = 50, CRs *n* = 48) group. Individually tailored nutritional guidance targeted to FCs was given to an intervention group by a clinical nutritionist. The main outcomes were dietary intake (3-day food record).

**Results:**

After the 6-month intervention, 63 FCs and 59 CRs in the intervention group and 50 FCs and 48 CRs in the control group were analyzed. In the intervention group of FCs, the intakes of protein, riboflavin, calcium, potassium, phosphorus, and iodine differed significantly (*p* < 0.05) compared to the control group. In addition, the intake of vitamin D supplementation improved in the intervention group of the FCs and CRs (*p* < 0.001).

**Conclusion:**

Individually tailored nutrition guidance improves the intake levels of crucial nutrients, such as the intake levels of protein, vitamin D, and calcium of the FCs. Further studies are warranted to optimize the methods to improve the nutrition of FCs.

**Registration number of Clinical Trials**: ClinicalTrials.gov NCT04003493 (1 July 2019).

## Introduction

Older family caregivers (FCs) and their care recipients (CRs) are threatened with poor nutritional status [1–4] and insufficient dietary intake [3]. FCs are also more vulnerable to the risk of malnutrition than other community-dwelling older people without a CR [5]. Insufficient dietary intake is common among older FCs [6, 7], although sufficient dietary intake has many benefits for older people. For example, optimal intake of nutrients can prevent frailty [8], and optimal protein intake can prevent the decline of physical performance [9] and improve lean body mass [10–13]. Because of the negative effects of poor nutritional status on many health outcomes, such as physical function [7, 14–16], cognitive status [15, 17], hospitalization [17], morbidity [7], and mortality [16], it is important to prevent deterioration of the nutritional status of older FCs, contributing their ability to serve as FCs. In addition to malnutrition, obesity is common in older community-dwelling people [18, 19]. Despite obesity, older people may have functional disability [18]. Obesity has many detrimental effects on health, such as risk of frailty [19], increased risk of falls [20], and declined cognition [21].

There has been only one earlier study with nutritional guidance and its impact on the dietary intake of older FCs [22]. In that earlier study, older FCs, especially male FCs, increased their energy and protein intake. Most earlier nutritional guidance or education trials with FCs and CRs have concentrated on studying changes in health outcomes and dietary intake of CRs [23–25]. These studies have shown that nutritional guidance can improve dietary intake and/or reduce the nutritional risk of CRs [23–25]. It has been found that malnourished and/or frail older people benefit from protein supplementation [12, 13]. However, there is also some evidence that non-frail community-dwelling older people did not benefit from protein supplementation [26].

To our knowledge, there are no randomized controlled trials about individually tailored nutritional guidance targeted to older FCs and its impact on the nutritional status of older FCs. The aim of this study was to assess the impact of individually tailored nutritional guidance on the dietary intake and nutritional status of older FCs and their CRs’ nutritional status.

## Materials and methods

### Study design

Lifestyle, Nutrition and Oral Health in caregivers (LENTO) is a randomized, controlled, population-based, nutritional, and oral health care intervention study of older caregivers aged 60 and above living in the town of Kuopio and the municipality of Vesanto (Eastern Finland) [27]. This study refers only to the nutritional intervention. The study was approved by the Ethics Committee, Hospital District of Northern Savo (No. 171/2019). All the participants gave written informed consent to participate in the study. The study was registered at ClinicalTrials.gov (NCT04003493, registered 1 July 2019). The Consolidated Standards of Reporting Trials (CONSORT) statement was followed in the reporting randomized controlled trial [28].

### Participants

**Fig. 1:**
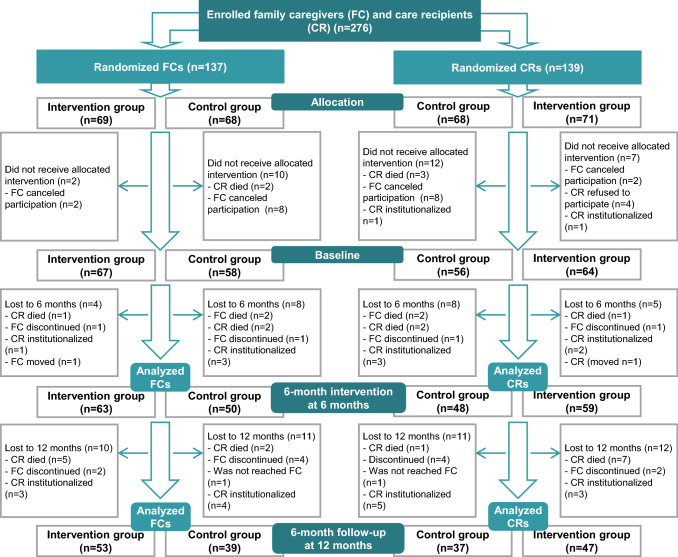
Flow chart of the study population

The study participants consisted of 113 FCs and 107 CRs living in the town of Kuopio and the municipality of Vesanto (Fig. 1). The study consisted of a lower number of CRs than FCs since some CRs refused to participate, died or were institutionalized, and only FCs of them wanted to participate. In addition, two FCs had two CRs (Fig. 1). Participants were recruited from FC registers of Kuopio and Vesanto between April 2019 and October 2019. The recruitment process of this study has been described previously [7]. All the participants had a valid care allowance (CA) during their participation in the 6-month intervention (6 months) and the 6-month follow-up (12 months) between June 2019 and December 2020. In Finland, CA includes benefits to the FC, such as taxable fees and 3 days of leave per month, and it is granted by municipality. FCs whose CR was receiving end-of-life care at baseline were excluded from the study. Because the FC registers of municipalities retrieve the age of the CR only, the FCs included in the study were 60 years old or above, except two of them who were under the age of 60. The CRs included in the study were 65 years old or above, except three of them who were under the age of 65, but they had been diagnosed with age-related disease of older people and belonged to the FC registers of older people.

### Study protocol and nutritional intervention

The participants were randomly computer assigned to an intervention group or a control group after enrolment without any criteria for the FCs or CRs. IBM SPSS Statistics software (v. 27, IBM Corp., Armonk, NY) was used in randomization. The allocation ratio was 1:1. The dropout rates were 9.6% in FCs and 10.8% in CRs (Fig. 1) during the 6-month intervention, and 26.4% in FCs and 30.0% in CRs from baseline to 12 months. Participants and researchers were not blinded because of practical impossibilities considering the design of the study.

**Fig. 2:**
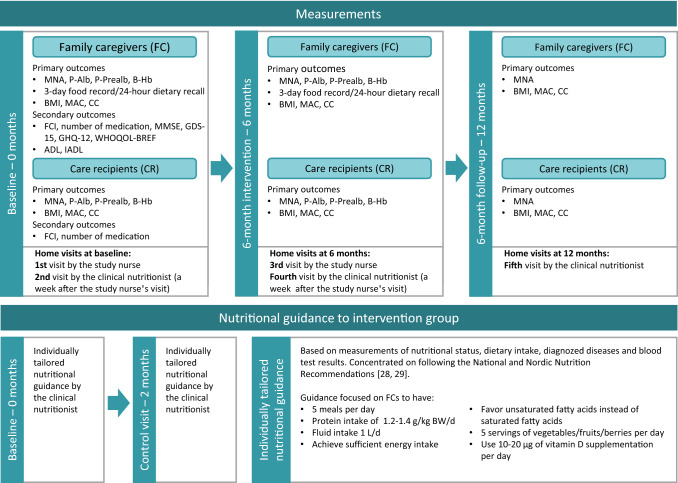
Flow chart of the measurements of the family caregivers (FC) and care recipients (CR), and individually tailored nutritional guidance

Both FCs and CRs were interviewed and measured during five home visits at three different time points (baseline, 6 months and 12 months) (Fig. 2). At baseline (0 months), the first home visit was made by a study nurse, followed by a visit by the clinical nutritionist a week later. The home visits at 6 months (6-month intervention) were carried out similarly to the baseline home visits with weekly visits by the study nurse and the clinical nutritionist (between December 2019 and 16 March 2020). Because of the COVID-19 pandemic, some of the participants had only one home visit at 6 months by the study nurse, who made all the measurements, and all the interviews were done by phone by the clinical nutritionist (between 17 March 2020 and June 2020). The home visit at 12 months (6-month follow-up) was made by the clinical nutritionist, who made all the measurements, and the interviews at 12 months were made by phone by the clinical nutritionist because of the COVID-19 pandemic (between June 2020 and December 2020). Only necessary measurements were made at FCs’ homes with their permission. Personal protective equipment and safety clearance were used during the home visits during the COVID-19 pandemic. The clinical nutritionist visited/contacted participants in the intervention group five times (baseline, 2 months, 6 months and two times at 12 months), and participants in the control group four times (baseline, 6 months and two times at 12 months).

Individually tailored nutritional guidance was given during two home visits at two different time points by a clinical nutritionist (baseline and 2 months) (Fig. 2). Individually tailored nutritional guidance was based on measurements of nutritional status, dietary intake, diagnosed diseases, and blood test results. Individually tailored nutritional guidance was based on the National and Nordic Nutrition Recommendations [29, 30]. Detailed information on the goals of the individually tailored nutritional guidance is presented in Fig. 2.

## Measurements

### Primary outcomes

Detailed information of measurements of the LENTO study have been described in a previous study [27]. Nutritional status was assessed with the Mini-Nutritional Assessment (MNA) [31, 32] by a clinical nutritionist, and part of the anthropometrics of the MNA by the study nurse at 6 months because of the COVID-19 pandemic. A study nurse drew blood samples for measurements of concentrations of plasma albumin (P-Alb), plasma prealbumin (P-Prealb), and blood hemoglobin (B-Hb). For anthropometric measurements, body weight, height, body mass index (BMI), mid-arm circumference (MAC) (measured from the middle of the upper arm of the non-dominant arm, relaxing next to the torso), and calf circumference (CC) (measured from the thickest point of the calf of the weaker side when sitting) were measured/calculated by the clinical nutritionist or the study nurse. Dietary intake was assessed with the 3-day food record, which was checked by the clinical nutritionist at return. For those who had not kept the food record, the clinical nutritionist performed a 24-h dietary recall (at baseline *n* = 15 (13.5% of the FCs), at 6 months *n* = 12 (10.8% of the FCs). Dietary intake was calculated with the AivoDiet software (version 2.2.0.0, AivoDiet by Mashie, Turku, Finland). Intake of energy, protein, carbohydrates, fiber, fat, saturated fat, monounsaturated fat, polyunsaturated fat, vitamin A, vitamin D, vitamin C, thiamine, riboflavin, niacin, vitamin B6, vitamin B12, folate, magnesium, calcium, iron, potassium, iodine, phosphorus, selenium, and zinc were calculated. Portion sizes were assessed by home dimensions and volumes of pieces. The clinical nutritionist also had a picture book of portions as an aid when checking portion sizes. Use of vitamin D supplementation (portion and frequency) was checked by the clinical nutritionist.

### Secondary outcomes

The study nurse determined comorbidities using a modified version of Functional Comorbidity Index (FCI) [33, 34], medication use based on daily prescription medication and nutrient supplement use, cognitive status with the Mini-Mental State Examination (MMSE) [35], depressive symptoms with Geriatric Depression Scale (GDS-15) [36], psychological distress with General Health Questionnaire (GHQ-12) [37], quality of life with The World Health Organization Quality of Life Brief Version (WHOQOL-BREF) [38], activities of daily living (ADL) with the Barthel index [39], and instrumental activity (IADL) with the Lawton and Brody Scale [40].

All the outcomes were performed on the FCs at baseline and 6 months, except for FCI and use of medication only at baseline (Fig. 2). The MNA and anthropometrics were performed on the FCs at 12 months. For CRs, FCI and use of medication were documented at baseline, MNA and anthropometrics were performed at baseline, 6 months, and 12 months, and biochemistry was measured at baseline and at 6 months.

### Statistical analyses

The sample size was based on plasma albumin concentration, with a 20% difference in plasma albumin concentration between the intervention and the control groups (power 0.80 and *p* value 0.05). Therefore, a sample size of 128 (*n* = 64 per group) was needed to demonstrate a statistically significant difference between the intervention and control groups.

A per protocol approach was used in the statistical analyses. Means with SDs or numbers with percentages were calculated from the baseline characteristics. Differences in baseline characteristics between the intervention and control groups were analyzed by independent samples *t*-tests (normally distributed outcomes), Mann–Whitney *U* tests (non-normally distributed outcomes), or Pearson Chi-square tests. Differences between the groups during the 6-month intervention (6 months, FCs *n* = 63 and CRs *n* = 59 in the intervention group, FCs *n* = 50 and CRs *n* = 48 in the control group) and the 6-month follow-up (12 months, FCs *n* = 53 and CRs *n* = 47 in the intervention group, FCs *n* = 39 and CRs *n* = 37 in the control group) (Fig. 1) and differences within the groups between the baseline and the 6- and 12-month measures were analyzed by generalized estimating equations (GEE) (with a linear model for continuous variables and an ordinal logistic model for ordinal variables) adjusted for age and sex. A *p* value less than 0.05 was considered statistically significant. Data analyses were performed with IBM SPSS Statistics software (v. 27, IBM Corp., Armonk, NY).

## Results

### Baseline characteristics of participants

The baseline characteristics were similar in the intervention and control groups (Table 1). The mean age of FCs in the whole study population was 74.3 ± 7.1, and 73.5% were women. Eighty percent of the FCs had normal nutritional status (MNA scores ≥ 24), and 20% had a risk of malnutrition (MNA scores 17–23.5). The main chronic diseases of FCs were rheumatoid arthritis or another connective tissue disease (37.2%) and diabetes, mainly type 2 (19.5%). CRs’ mean age was 79.3 ± 7.9, and 33.6% were women. Sixty percent of the CRs had a risk of malnutrition, and 12% of the CRs had malnutrition. The main chronic diseases of the CRs were dementia (57.9%) and diabetes, mainly type 2 (33.6%).

**Table 1:** Baseline characteristics of the family caregivers (FC) and the care recipients (CR)

Characteristics	FCs			CRs		
	Intervention group (*n* = 63) Mean ± SD	Control group (*n* = 50) Mean ± SD	*P* value^a^	Intervention group (*n* = 59) Mean ± SD	Control group (*n* = 48) Mean ± SD	*P* value^a^
Age (year)	74.5 ± 6.4	74.0 ± 8.0	0.704^b^	79.6 ± 7.9	78.9 ± 7.9	0.653^b^
Females, *n *(%)	45 (71.4)	38 (76.0)	0.585^c^	22 (37.3)	14 (29.2)	0.377^c^
MNA scores	25.4 ± 1.9	25.5 ± 2.0	0.984	21.5 ± 3.7	21.9 ± 3.0	0.484^b^
Normal nutritional status, *n *(%)	51 (81.0)	39 (78.0)	0.699^c^	17 (28.8)	16 (33.3)	0.238^c^
Risk of malnutrition, *n* (%)	12 (19.0)	11 (22.0)		36 (61.0)	31 (64.6)	
P-Alb (g/L)^d^	37.5 ± 2.3	37.6 ± 2.4	0.948	34.8 ± 3.5	34.0 ± 3.4	0.316
P-Prealb (g/L)^e^	0.25 ± 0.04	0.24 ± 0.05	0.431^b^	0.23 ± 0.04	0.23 ± 0.05	0.578
B-Hb (g/L)^f^	135.8 ± 10.5	134.7 ± 10.5	0.607^b^	136.4 ± 13.4	134.7 ± 4.3	0.973
BMI (kg/cm^2^)^g^	29.3 ± 6.3	27.4 ± 4.7	0.113	29.0 ± 7.0	27.6 ± 4.3	0.723
FCI	2.0 ± 1.6	2.0 ± 1.4	0.631	3.5 ± 2.0	3.5 ± 2.0	0.819
Number of medication	5.6 ± 4.1	5.1 ± 3.3	0.506	8.5 ± 4.2	8.8 ± 4.5	0.841
MMSE	26.1 ± 3.2	26.9 ± 2.8	0.552			
GDS-15	3.1 ± 2.3	2.8 ± 2.7	0.309			
GHQ-12	12.5 ± 5.1	12.0 ± 5.6	0.454			
WHOQOL-BREF	94.0 ± 9.4	94.0 ± 9.8	0.869^b^			
ADL	97.9 ± 3.3	98.3 ± 3.7	0.606			
IADL	7.8 ± 0.6	7.9 ± 0.5	0.199			

*FC*  family caregiver, *CR* care recipient, *SD* standard deviation, *MNA* Mini-Nutritional Assessment, *BMI* body mass index, *B-Hb* blood haemoglobin, *P-Alb* plasma albumin, *P-Prealb* plasma prealbumin, *FCI *functional comorbidity index, *MMSE* Mini-Mental State Examination, *GHQ*-12 General Health Questionnaire, *WHOQOL-BREF* World Health Organization Quality of Life Brief Version, *ADL* Barthel index, *IADL* Instrumental activities of daily living

^a^Difference between groups with Mann–Whitney’s *U* test (non-normally distributed outcomes)

^b^Difference between groups with independent samples *T*-test (normally distributed outcomes)

^c^Difference between groups with Pearson Chi-square

^d^Intervention group FCs *n* = 60, CRs *n* = 49, control group FCs *n* = 47, CRs *n* = 42

^e^Intervention group FCs *n *= 60, CRs *n* = 45, control group FCs *n* = 48, CRs *n* = 39

^f^Intervention group FCs *n* = 59, CRs *n* = 53, control group FCs *n* = 49, CRs *n* = 44

^g^Intervention group FCs *n* = 60, CRs *n* = 46, control group FCs *n* = 47, CRs *n* = 39

### Changes in dietary intake

Changes in dietary intake of the FCs during the 6-month intervention are presented in Table 2. There was a significant difference between the groups (*p* = 0.001) in protein intake (*E*%). The intervention group improved their mean protein intake (16.3 ± 2.9 vs. 17.6 ± 2.7 *E*%, *p* < 0.001). A difference between the groups was observed also in mean protein intake per kg BW (*p* = 0.015) during the 6-month intervention. The intervention group increased their mean protein intake per kg BW (0.95 ± 0.34 vs. 1.05 ± 0.37 g/kg BW/d, *p* = 0.002). Regarding the intake of vitamins, the only difference between the groups was in riboflavin intake (*p* = 0.010) during the 6-month intervention, the intervention group increased their riboflavin intake significantly (*p* = 0.007).

**Table 2:** Changes in dietary intake of the family caregivers (FC) during the 6-month intervention

	Intervention group (*n *= 62)			Control group (*n* = 49)			Time × group interaction between the groups
	0 months	6 months	*P* value^a^	0 months	6 months	*P* value^a^	*P* value^a^
	Mean ± SD	Mean ± SD		Mean ± SD	Mean ± SD		
Energy (kcal/day)	1763 ± 491	1781 ± 427		1646 ± 339	1670 ± 364		0.316
Protein (g/day)	71.2 ± 21.9	78.2 ± 21.1	0.001	67.8 ± 16.5	66.2 ± 14.6	0.608	0.001
Protein (g/kg BW/day)^b^	0.95 ± 0.34	1.05 ± 0.37	0.002	0.98 ± 0.27	0.95 ± 0.26	0.381	0.015
Protein (*E*%)	16.3 ± 2.9	17.6 ± 2.7	< 0.001	16.5 ± 2.7	16.1 ± 3.0	0.344	0.001
Carbohydrates (*E*%)	45.3 ± 6.4	45.8 ± 6.3		44.6 ± 5.3	45.8 ± 5.9		0.570
Fiber (g/day)	20.1 ± 8.2	20.9 ± 8.2		19.0 ± 5.0	19.6 ± 5.0		0.400
Fat (*E*%)	34.1 ± 6.3	32.7 ± 6.2		34.9 ± 5.2	34.2 ± 5.7		0.163
SFA (*E*%)	13.0 ± 3.8	12.2 ± 3.5		12.5 ± 3.1	12.4 ± 3.3		0.408
MUFA (*E*%)	11.8 ± 2.5	11.6 ± 2.7		12.3 ± 2.5	12.1 ± 2.5		0.521
PUFA (*E*%)	5.8 ± 1.6	5.6 ± 1.8		6.3 ± 1.7	5.9 ± 1.6		0.158
Vitamin A (µg/day)	700 ± 358	783 ± 769		840 ± 552	942 ± 790		0.073
Vitamin D (µg/day)	13.3 ± 6.6	11.9 ± 4.8		11.3 ± 6.0	10.5 ± 3.8		0.071
Vitamin C (mg/day)	127 ± 85	132 ± 73		124 ± 53	117 ± 57		0.613
Thiamine (mg/day)	1.3 ± 0.5	1.4 ± 0.4		1.3 ± 0.4	1.3 ± 0.3		0.125
Riboflavin (mg/day)	1.9 ± 0.7	2.0 ± 0.6	0.007	1.8 ± 0.6	1.7 ± 0.5	0.541	0.010
Niacin (mg/day)	29 ± 9.7	30 ± 8.6		28 ± 7.2	27 ± 7.0		0.101
Vitamin B6 (mg/day)	1.9 ± 0.8	2.0 ± 1.0		1.8 ± 0.6	1.6 ± 0.6		0.099
Vitamin B12 (µg/day)	5.4 ± 3.1	6.0 ± 3.4		5.3 ± 3.5	5.6 ± 3.6		0.580
Folate (µg/day)	258 ± 93	284 ± 99		268 ± 81	272 ± 91		0.071
Magnesium (mg/day)	349 ± 101	363 ± 93		340 ± 61	333 ± 63		0.114
Calcium (mg/day)	1097 ± 409	1163 ± 363	0.102	1007 ± 394	934 ± 337	0.213	0.007
Iron (mg/day)	10.6 ± 3.7	10.6 ± 3.3		10.7 ± 3.7	11.1 ± 3.9		0.734
Potassium (mg/day)	3723 ± 1040	3818 ± 965	0.261	3639 ± 632	3415 ± 697	0.023	0.029
Iodine (µg/day)	231 ± 78	249 ± 67	0.016	218 ± 60	209 ± 58	0.318	0.005
Phosphorus (mg/day)	1484 ± 469	1575 ± 427	0.013	1400 ± 340	1363 ± 338	0.543	0.010
Selenium (µg/day)	63 ± 21	69 ± 28		60 ± 16	59 ± 15		0.111
Zinc (mg/day)	11.1 ± 3.4	11.1 ± 3.2		10.4 ± 2.5	10.3 ± 2.2		0.413

*FC* family caregiver, *SD* standard deviation, *SFA* saturated fatty acids, *MUFA* monounsaturated fatty acids

^a^Difference between groups, and between 0- and 6-month measures by generalized estimated equations adjusted with age and gender

^b^Intervention group *n* = 60, control group *n* = 47

Regarding the intake of minerals, there were significant differences between the groups in the intake of calcium (*p* = 0.007), potassium (*p* = 0.029), iodine (*p* = 0.005), and phosphorus (*p* = 0.010) during the 6-month intervention (Table 2). The intakes of iodine (*p* = 0.016) and phosphorus (*p* = 0.013) increased significantly in the intervention group. The intake of potassium decreased in the control group during the 6-month intervention (*p* = 0.023).

Changes in the use of vitamin D supplementation differed significantly between the intervention and the control groups in both the FCs and the CRs during the 6-month intervention (< 0.001) (Table 3). Both the FCs and the CRs in the intervention group increased their use of vitamin D supplementation (*p* < 0.001). At baseline, even one-third of the participants in the intervention group had no vitamin D supplementation, or it was less than 10 µg/day. During the 6-month intervention, 97% of the FCs and 95% of the CRs had vitamin D supplementation of 10 µg/day or higher. In the control group, there were no significant changes in the use of vitamin D supplementation in the FCs or the CRs during the 6-month intervention.

**Table 3:** Changes in vitamin D supplementation of the family caregivers (FC) and care recipients (CR) during the 6-month intervention

	Intervention group (FCs *n* = 63, CRs *n* = 59)			Control group (FCs n = 50, CRs n = 48)			Time × group interaction between the groups
	0 months	6 months	p-value^a^	0 months	6 months	*P* value^a^	*P* value^a^
	*n* (%)	*n* (%)		*n* (%)	*n* (%)		
FCs			< 0.001			0.195	< 0.001
0–9 µg/day	21 (33.3)	2 (3.2)		15 (30.0)	11 (22.0)		
10–19 µg/day	10 (15.9)	8 (12.7)		12 (24.0)	8 (16.0)		
20–29 µg/day	24 (38.1)	37 (58.7)		11 (22.0)	17 (34.0)		
≥ 30 µg/day	8 (12.7)	16 (25.4)		12 (24.0)	14 (28.0)		
CRs			< 0.001			0.168	< 0.001
0–9 µg/day	18 (30.5)	3 (5.1)		17 (35.4)	13 (27.1)		
10–19 µg/day	8 (13.6)	6 (10.2)		6 (12.5)	3 (6.3)		
20–29 µg/day	26 (44.1)	35 (59.3)		19 (39.6)	23 (47.9)		
≥ 30 µg/day	7 (11.9)	15 (25.4)		6 (12.5)	9 (18.8)		

*FC* family caregiver, *CR* care recipient

^a^ Difference between groups, and between 0- and 6-month measures by generalized estimated equations adjusted with age and gender

### Changes in the nutritional status of the FCs

There were no significant differences in the nutritional status of the FCs (Table 4) during the 6-month intervention. A difference approaching statistical significance between the groups in biochemistry was observed in blood hemoglobin of the FCs (*p* = 0.057) during the 6-month intervention (Table 4). The intervention group retained their hemoglobin level, while it decreased in the control group (*p* = 0.018) (not shown in a table). In anthropometrics, there were significant differences between the groups in MAC (*p* < 0.001) and CC (*p* = 0.020) of the FCs, with a significant decrease in MAC (*p* = 0.001) in the intervention group.

**Table 4:** Changes in nutritional status, biochemistry, and anthropometrics of the family caregivers (FC) during the 6-month intervention

	Intervention group (*n* = 63)			Control group (*n* = 50)			Time × group interaction between the groups
	0 months	6 months	*P *value^a^	0 months	6 months	*P *value^a^	*P* value^a^
	Mean ± SD	Mean ± SD		Mean ± SD	Mean ± SD		
MNA scores	25.4 ± 1.9	25.5 ± 2.1		25.5 ± 2.0	25.5 ± 2.1		0.982
P-Alb (g/L)^b^	37.4 ± 2.3	37.2 ± 2.8		37.6 ± 2.4	37.5 ± 2.8		0.730
P-Prealb (g/L)^c^	0.25 ± 0.04	0.25 ± 0.05		0.24 ± 0.05	0.25 ± 0.05		0.558
B-Hb (g/L)^d^	136 ± 11	135 ± 11		135 ± 11	132 ± 11		0.057
BMI (kg/cm^2^)^b^	29.2 ± 5.9	29.1 ± 5.9		27.3 ± 4.7	27.4 ± 4.8		0.127
MAC (cm)^b^	33.2 ± 4.3	32.7 ± 4.4	0.001	31.7 ± 4.1	31.4 ± 3.7	0.159	< 0.001
CC (cm)^b^	39.1 ± 3.7	38.8 ± 3.9	0.086	37.5 ± 3.5	37.4 ± 3.8	0.359	0.020

*FC* family caregiver, *SD* standard deviation, *MNA* Mini-Nutritional Assessment, *B-Hb* blood haemoglobin, *P-Alb* plasma albumin, *P-Prealb* plasma prealbumin, *BMI* body mass index, *MAC* mid-arm circumference, *CC* calf circumference

^a^ Difference between groups, and between 0- and 6-month measures by generalized estimated equations adjusted with age and gender

^b^ Intervention group *n* = 60, control group *n* = 47

^c^ Intervention group *n* = 60, control group *n* = 48

^d^ Intervention group *n* = 59, control group *n* = 49

### Changes in the nutritional status of the CRs

There were no significant differences in the nutritional status of the CRs (Table 5) during the 6-month intervention. Examining differences between the three time points of the study (0 months, 6 months, 12 months) over the 12-month period (intervention group *n* = 47, control group *n* = 37) (Fig. 1), there was a significant difference in MNA scores between the groups in the CRs (*p* < 0.001) (not shown in a table). Differences in the intervention group were significant between the baseline and at 12 months (*p* = 0.049), when the mean MNA score increased from the baseline to the 12-month time point (21.9 ± 3.4 vs. 22.7 ± 2.5). The same improvement in the mean MNA score was observed in the control group between the baseline and the 12-month time point (*p* = 0.003) and at the 6- and 12-month time points (*p* = 0.003) (21.6 ± 3.2 vs. 21.5 ± 3.8 vs. 22.9 ± 2.3).

**Table 5:** Changes in nutritional status, biochemistry, and anthropometrics of the care recipients (CR) during the 6-month intervention

	Intervention group (*n* = 59)			Control group (*n* = 48)			Time × group interaction between the groups
	0 months	6 months	*P *value^a^	0 months	6 months	*P* value^a^	*P *value^a^
	Mean ± SD	Mean ± SD		Mean ± SD	Mean ± SD		
MNA scores	21.5 ± 3.7	21.5 ± 3.7		21.9 ± .3.0	21.4 ± 3.5		0.660
P-Alb (g/L)^b^	34.9 ± 3.4	34.7 ± 3.2		34.1 ± 3.5	34.3 ± 4.1		0.624
P-Prealb (g/L)^c^	0.23 ± 0.04	0.23 ± 0.04		0.23 ± 0.05	0.23 ± 0.06		0.512
B-Hb (g/L)^d^	136 ± 14	135 ± 16		135 ± 16	132 ± 18		0.133
BMI (kg/cm^2^)^e^	28.5 ± 7.2	28.7 ± 6.8		27.8 ± 4.5	27.8 ± 4.5		0.407
MAC (cm)^f^	32.0 ± 5.2	31.8 ± 4.9	0.240	31.7 ± 3.9	31.0 ± 4.0	0.001	0.007
CC (cm)^f^	37.3 ± 5.0	37.2 ± 4.7		36.0 ± 3.4	35.6 ± 3.8		0.225

*CR* care recipient, *SD* standard deviation, *MNA* Mini-Nutritional Assessment, *B-Hb* blood haemoglobin, *P-Alb* plasma albumin, *P-Prealb* plasma prealbumin, *BMI* body mass index, *MAC* mid-arm circumference, *CC* calf circumference

^a^ Difference between groups, and between 0- and 6-month measures by generalized estimated equations adjusted with age and gender

^b^ Intervention group *n* = 49, control group *n* = 42

^c^ Intervention group *n* = 45, control group *n* = 39

^d^ Intervention group *n* = 53, control group *n* = 44

^e^ Intervention group *n* = 46, control group *n* = 39

^f^ Intervention group *n* = 49, control group *n* = 44

There were no significant differences between the groups in the biochemistry of the CRs during the 6-month intervention (Table 5). The MAC of the CRs differed significantly between the groups (*p* = 0.007) at the end of the 6-month intervention. MAC decreased (*p* = 0.001) in the control group, while no significant change was observed in the intervention group.

## Discussion

Individually tailored nutritional guidance improved the intake of protein, riboflavin, iodine, and phosphorus of the FCs during the 6-month intervention. Significant differences between the intervention and control groups were observed in the intake levels of protein, riboflavin, calcium, potassium, phosphorus, and iodine during the 6-month intervention. In addition, the intake of vitamin D supplementation improved in the intervention group for both the FCs and the CRs. The MNA scores of the FCs and the CRs did not change during the 6-month intervention in either of the groups. However, there were an increase during the 12-month period, i.e. the 6-month intervention and the 6-month follow-up, in the MNA scores of the CRs in the intervention group, but there was also an increase in the CRs in the control group when 12-month results were compared with 0- and 6-month time points.

The main result of this study is the increased protein intake of the FCs in the intervention group compared with the control group. Previous studies with older people have shown that the consumption of dairy products, eggs, and fish can increase due to nutritional intervention [41, 42]. When reflecting on what kind of changes the FCs of this study made to improve their protein intake, it seems that the improved protein intake of the FCs in the intervention group was mainly due to increased consumption of dairy products. The increased intakes of riboflavin, iodine, and phosphorus in the intervention group, and the increased difference between groups in the intakes of calcium and potassium supports an increased use of dairy products. The facility, familiarity, and likability of dairy products can be important reasons why FCs increased their use. In addition, there are many protein-rich dairy products available in Finland, which are easy to add to the diet. Despite the increased protein intake, the mean protein intake (1.05 ± 3.7 g/kg BW/day) of the FCs in the intervention group did not reach the recommended intake (1.2–1.4 g/kg BW/day) according to the Nordic Nutrition Recommendations [30]. Similar findings showed Kunvik et al. [22] with older FCs when protein intake increased + 0.1 g/kg BW/day to 0.96 g/kg BW/day in the intervention group. Lower than recommended protein intake may be due to the inability of FCs to improve the quality or number of their main meals. More attention to cooking can be experienced burdensome and they have chosen easier ways (dairy products) to improve their protein intake. However, this has not been sufficient for all FCs. The burden of care may have affected unaccomplished changes in nutrition. Nutritional guidance can prevent insufficient protein intake in older people and consequently protects against weight loss [43], prevents frailty [44], predicts better physical performance [45], and prevents mobility limitations [46]. In most of the earlier intervention studies with positive effects of higher protein intake or protein supplementation on lean body mass, an increase in physical activity was also included, so it is difficult to draw specific conclusions regarding protein intake alone [10, 11]. Caregivers have limited time to have physical activity because of hours of care [6], and synergy of optimal nutrition and physical activity can be missing.

Considering of the intakes of vegetables, fruits, or berries, it seems that FCs could not considerably improve their intake of vegetables, fruits, or berries. This is supported by the results that there were no significant changes in e.g. vitamin C, folate or fiber. Previously, Berendsen et al. [41] and Bernstein et al. [47] showed that older people can improve their fruit and vegetable intake due to nutritional guidance. However, both these earlier studies evaluated home-dwelling older people [41, 47], and counseling was given nine times during a 12-month intervention [41] or through eight home visits, biweekly phone contacts, and monthly letters during a 6-month intervention [47]. The present study consists of older FCs who are more vulnerable to the risk of malnutrition than home-dwelling older people [5], and they had nutritional guidance only twice during the 6-month intervention. Moreover, some of the participants ended the 6-month intervention during the first wave of the COVID-19 pandemic. Voluntary isolation was recommended for older people at that time by the Finnish government. This could have affected the shopping behaviors of older people, i.e., they might have visited grocery stores less often or not at all, relatives or friends may have taken care of their food purchases, or FCs have made grocery shopping online. All these factors could have decreased the consumption of fresh products, such as vegetables, fruits, and fresh fish, at the 6-month time point, which was observed earlier in one study with adults [48].

Our study confirms an earlier finding that nutritional guidance improves the use of vitamin D supplementation in older people [41]. Vitamin D deficiency is associated with many health outcomes, such as bone density [49], sarcopenia [50], and poor physical performance [51, 52] in older people. Therefore, it is important and efficient to take sufficient vitamin D supplementation with nutritional guidance.

Although nutrient intake of the FCs improved in the intervention group, it was not seen as an improvement in the MNA scores. However, it had a positive impact on blood hemoglobin concentration in the intervention group. There were significant differences between the groups in the MAC and CC of the FCs, and the MAC of the CRs. However, these changes were minor and no conclusions about the effect of nutritional guidance to the MAC and CC cannot be drawn. The study sample consisted mainly of FCs with normal nutritional status (79.6%), partly explaining the lack of improvement in the MNA scores. FCs also have a demanding duty to take care of their CRs. This can be seen as increases in stress [53], caregiver burden [54], and sleeping disturbances, mostly because of care performed at night [55]. All these factors have a negative effect on nutritional status [56–58].

Earlier evidence of the effectiveness of nutritional guidance targeted to FCs on the nutritional status of CRs is not clear. Fernández-Barrés et al. [23] showed that it has a positive impact on the nutritional status of CRs, while Shatenstein et al. [24] did not find any effect. In the present study, there was some evidence of positive effects of nutritional guidance on CRs’ MNA scores in the intervention group. However, there were parallel changes in the MNA scores of the control group. Even two-thirds of the CRs in the control group were malnourished or at risk of malnutrition at baseline, and almost all of them were guided to the health care or received nutritional guidance from the clinical nutritionist if necessary because of the ethical approval. This could have protected CRs in the control group from declined nutritional status during the 6-month intervention and may explain the increase in the MNA scores at the 12-month time point.

It seems that FCs can easily adopt improvements by simple means in their dietary intake and use of supplementations, but because of daily tasks and care routines, some more time-consuming changes are more difficult to adopt [55]. This can prevent more substantial improvements and positive impacts on the nutritional status of FCs and CRs. They can also need more frequent support and monitoring. For example, in a Spanish study, CRs’ nutritional status increased during the intervention, which included monthly support and dietary advice about optimal nutrition by nurses [23]. There was also more frequent support and guidance in the two other previous studies [41, 47]. In addition, van den Helder et al. [42] reported higher protein intakes after one group session, monthly face-to-face sessions, and weekly/monthly video sessions with a dietician.

The intervention increased protein intake, although it did not reach recommendations [30]. It is important to be able to prevent decrease in dietary intake, not only to improve it, because decreased dietary intake can accelerate the deterioration of health. ESPEN guidelines recommend that protein intake of older people should be at least 1 g/kg BW/day until more evidence is available [59]. This recommendation was achieved in the present study, so this could protect FCs of negative health effects of protein intake being too low (< 1 g/kg BW/day). Nutritional guidance should be part of the services to FCs, preventing early deterioration of nutritional status and promoting the ability to serve as FCs. However, further studies are warranted to optimize the support for FCs’ nutritional status.

The strengths of this study are the randomized, population-based design and the validated methods used and suitable in older people. The data on MNA and food records were collected/checked by a clinical nutritionist, which improves the reliability of these tools. However, under- or over-reporting assessing dietary intake with the food record or 24-h recall is possible. Our study was carried out during home visits and phone calls, which improved FCs’ ability to participate. The COVID-19 pandemic could have changed the behavior of older people or increased their anxiety during this intervention, and some benefits of the intervention could have been diluted.

## Conclusions

Individually tailored nutritional guidance improves intake levels of crucial nutrients among FCs, such as intake levels of protein, vitamin D, and calcium without a significant improvement in the MNA scores. Further studies are warranted to optimize the methods to improve the nutritional status of FCs.

## References

[1] Rullier L, Lagarde A, Bouisson J, Bergua V, Barberger-Gateau P (2013). Nutritional status of community-dwelling older people with dementia: associations with individual and family caregivers' characteristics. Int J Geriatr Psychiatry.

[2] Tombini M, Sicari M, Pellegrino G, Ursini F, Insardá P, Di Lazzaro V (2016). Nutritional status of patients with Alzheimer's disease and their caregivers. J Alzheimers Dis.

[3] Puranen TM, Pietila SE, Pitkala KH, Kautiainen H, Raivio M, Eloniemi-Sulkava U, Jyväkorpi SK, Suominen M (2014). Caregivers' male gender is associated with poor nutrient intake in AD families (NuAD-trial). J Nutr Health Aging.

[4] Torres SJ, McCabe M, Nowson CA (2010). Depression, nutritional risk and eating behaviour in older caregivers. J Nutr Health Aging.

[5] Leij-Halfwerk S, Verwijs MH, van Houdt S, Borkent JW, Guaitoli PR, Pelgrim T, Heymans MW, Power L, Visser M, Corish CA, de van der Schueren MAE.  (2019). Prevalence of protein-energy malnutrition risk in European older adults in community, residential and hospital settings, according to 22 malnutrition screening tools validated for use in adults ≥65 years: a systematic review and meta-analysis. Maturitas.

[6] Snyder SA, Vitaliano PP (2020). Caregiver psychological distress: Longitudinal relationships with physical activity and diet. Am J Alzheimers Dis Other Demen.

[7] Koponen S, Nykänen I, Savela R, Välimäki T, Suominen AL, Schwab U (2021). Inadequate intake of energy and nutrients is common in older family caregivers. Nutrients.

[8] Moradell A, Fernández-García ÁI, Navarrete-Villanueva D, Sagarra-Romero L, Gesteiro E, Pérez-Gómez J, Rodríguez-Gómez I, Ara I, Casajús JA, Vicente-Rodríguez G, Gómez-Cabello A (2021). Functional frailty, dietary intake, and risk of malnutrition are nutrients involved in muscle synthesis the key for frailty prevention?. Nutrients.

[9] McLean RR, Mangano KM, Hannan MT, Kiel DP, Sahni S (2016). Dietary protein intake is protective against loss of grip strength among older adults in the framingham offspring cohort. J Gerontol A Biol Sci Med Sci.

[10] Ten Haaf DSM, Eijsvogels TMH, Bongers CCWG, Horstman AMH, Timmers S, de Groot LCPGM, Hopman MTE (2019). Protein supplementation improves lean body mass in physically active older adults: a randomized placebo-controlled trial. J Cachexia Sarcopenia Muscle.

[11] Englund DA, Kirn DR, Koochek A, Zhu H, Travison TG, Reid KF, von Berens Å, Melin M, Cederholm T, Gustafsson T, Fielding RA (2017). Nutritional supplementation with physical activity improves muscle composition in mobility-limited older adults, the vive2 study: a randomized, double-blind, placebo-controlled trial. J Gerontol A Biol Sci Med Sci.

[12] Park Y, Choi J, Hwang H (2018). Protein supplementation improves muscle mass and physical performance in undernourished prefrail and frail elderly subjects: a randomized, double-blind, placebo-controlled trial. Am J Clin Nutr.

[13] Dewansingh P, Melse-Boonstra A, Krijnen WP, van der Schans CP, Jager-Wittenaar H, van den Heuvel EGHM (2018). Supplemental protein from dairy products increases body weight and vitamin D improves physical performance in older adults: a systematic review and meta-analysis. Nutr Res.

[14] Ge L, Yap CW, Heng BH (2020). Association of nutritional status with physical function and disability in community-dwelling older adults: a longitudinal data analysis. J Nutr Gerontol Geriatr.

[15] Bakhtiari A, Pourali M, Omidvar S (2020). Nutrition assessment and geriatric associated conditions among community dwelling Iranian elderly people. BMC Geriatr.

[16] Wei K, Nyunt MSZ, Gao Q, Wee SL, Ng TP (2019). Long-term changes in nutritional status are associated with functional and mortality outcomes among community-living older adults. Nutrition.

[17] Corish CA, Bardon LA (2019). Malnutrition in older adults: screening and determinants. Proc Nutr Soc.

[18] Gretebeck KA, Sabatini LM, Black DR, Gretebeck RJ (2017). Physical activity, functional ability, and obesity in older adults: a gender difference. J Gerontol Nurs.

[19] Yuan L, Chang M, Wang J (2021). Abdominal obesity, body mass index and the risk of frailty in community-dwelling older adults: a systematic review and meta-analysis. Age Ageing.

[20] Trevisan C, Crippa A, Ek S, Welmer A, Sergi G, Maggi S, Manzato E, Bea JW, Cauley JA, Decullier E, Hirani V, LaMonte MJ, Lewis CE, Schott A, Orsini N, Rizzuto D (2019). Nutritional status, body mass index, and the risk of falls in community-dwelling older adults: a systematic review and meta-analysis. J Am Med Dir Assoc.

[21] Dye L, Boyle NB, Champ C, Lawton C (2017). The relationship between obesity and cognitive health and decline. Proc Nutr Soc.

[22] Kunvik S, Valve R, Salonoja M, Suominen MH (2018). Does tailored nutritional guidance encourage older caregivers to increase their protein intake? The Care Nutrition Trial (RCT). J Aging Res Clin Practice.

[23] Fernández-Barrés S, García-Barco M, Basora J, Martínez T, Pedret R, Arija V (2017). The efficacy of a nutrition education intervention to prevent risk of malnutrition for dependent elderly patients receiving home care: A randomized controlled trial. Int J Nurs Stud.

[24] Shatenstein B, Kergoat M, Reid I (2017). Outcome of a targeted nutritional intervention among older adults with early-stage Alzheimer's disease: the nutrition intervention study. J Appl Gerontol.

[25] Suominen MH, Puranen TM, Jyväkorpi SK, Eloniemi-Sulkava U, Kautiainen H, Siljamäki-Ojansuu U, Pitkalä KH (2015). Nutritional guidance improves nutrient intake and quality of life, and may prevent falls in aged persons with Alzheimer disease living with a spouse (NuAD trial). J Nutr Health Aging.

[26] Ten Haaf DSM, Nuijten MAH, Maessen MFH, Horstman AMH, Eijsvogels TMH, Hopman MTE (2018). Effects of protein supplementation on lean body mass, muscle strength, and physical performance in nonfrail community-dwelling older adults: a systematic review and meta-analysis. Am J Clin Nutr.

[27] Nykänen I, Välimäki T, Suominen L, Schwab U (2021). Optimizing nutrition and oral health for caregivers — intervention protocol. Trials.

[28] Schulz KF, Altman DG, Moher D, the CONSORT Group (2010). CONSORT 2010 Statement: updated guidelines for reporting parallel group randomised trials. BMC Med.

[29] National Nutrition Council (2014) Finnish Nutritional Recommendations 2014. Helsinki

[30] Nordic Council of Ministers (2012). Nordic Nutrition Recommendations 2012.

[31] Guigoz Y (2006). The Mini Nutritional Assessment (MNA) review of the literature–What does it tell us?. J Nutr Health Aging.

[32] Guigoz Y, Lauque S, Vellas BJ (2002). Identifying the elderly at risk for malnutrition. The mini nutritional assessment. Clin Geriatr Med.

[33] Groll DL, To T, Bombardier C, Wright JG (2005). The development of a comorbidity index with physical function as the outcome. J Clin Epidemiol.

[34] Tikkanen P, Nykänen I, Lönnroos E, Sipilä S, Sulkava R, Hartikainen S (2012). Physical activity at age of 20–64 years and mobility and muscle strength in old age: a community-based study. The J of Gerontol: Ser A.

[35] Folstein MF, Folstein SE, McHugh PR (1975). “Mini-mental state”: A practical method for grading the cognitive state of patients for the clinician. J Psychiatr Res.

[36] Yesavage JA, Sheikh JI (1986). Geriatric Depression Scale (GDS). Clin Gerontol.

[37] Goldberg D (1972). The Detection of Psychiatric Illness by Questionnaire.

[38] The WHOQOL Group (1998). Development of the World Health Organization WHOQOL-BREF quality of life assessment. Psychol Med.

[39] Mahoney FI, Barthel DW (1965). Functional Evaluation: The barthel index. Md State Med J.

[40] Lawton MP, Brody EM (1969). Assessment of older people: self-maintaining and instrumental activities of daily living. Gerontologist.

[41] Berendsen AAM, van de Rest O, Feskens EJM, Santoro A, Ostan R, Pietruszka B, Brzozowska A, Stelmaszczyk-Kusz A, Jennings A, Gillings R, Cassidy A, Caille A, Caumon E, Malpuech-Brugere C, Franceschi C, de Groot LCPGM (2018). Changes in Dietary Intake and Adherence to the NU-AGE Diet Following a One-Year dietary intervention among european older adults-results of the nu-age randomized trial. Nutrients.

[42] van den Helder J, Verlaan S, Tieland M, Scholten J, Mehra S, Visser B, Kröse BJA, Engelbert RHH, Weijs PJM (2021). Digitally supported dietary protein counseling changes dietary protein intake, sources and distribution in community-dwelling older adults. Nutrients.

[43] Gray-Donald K, St-Arnaud-McKenzie D, Gaudreau P, Morais JA, Shatenstein B, Payette H (2014). Protein intake protects against weight loss in healthy community-dwelling older adults. J Nutr.

[44] Coelho-Júnior HJ, Rodrigues B, Uchida M, Marzetti E (2018). Low protein intake is associated with frailty in older adults: a systematic review and meta-analysis of observational studies. Nutrients.

[45] Coelho-Junior HJ, Marzetti E, Picca A, Cesari M, Uchida MC, Calvani R (2020). Protein intake and frailty: a matter of quantity, quality, and timing. Nutrients.

[46] Houston DK, Tooze JA, Garcia K, Visser M, Rubin S, Harris TB, Newman AB, Kritchevsky SB (2017). Protein intake and mobility limitation in community-dwelling older adults: the Health ABC study. J Am Geriatr Soc.

[47] Bernstein A, Nelson ME, Tucker KL, Layne J, Johnson E, Nuernberger A, Castaneda C, Judge JO, Buchner D, Singh MF (2002). A home-based nutrition intervention to increase consumption of fruits, vegetables, and calcium-rich foods in community dwelling elders. J Am Diet Assoc.

[48] Deschasaux-Tanguy M, Druesne-Pecollo N, Esseddik Y, de Edelenyi FS, Allès B, Andreeva VA, Baudry J, Charreire H, Deschamps V, Egnell M, Fezeu LK, Galan P, Julia C, Kesse-Guyot E, Latino-Martel P, Oppert J, Péneau S, Verdot C, Hercberg S, Touvier M (2021). Diet and physical activity during the coronavirus disease 2019 (COVID-19) lockdown (March-May 2020): results from the French NutriNet-Santé cohort study. Am J Clin Nutr.

[49] Lins Vieira NF, da Silva NJ, do Nascimento CQ, Barros Neto JA, Oliveira Dos Santo ACS,  (2021). Association between bone mineral density and nutritional status, body composition and bone metabolism in older adults. J Nutr Health Aging.

[50] Remelli F, Vitali A, Zurlo A, Volpato S (2019). Vitamin D deficiency and sarcopenia in older persons. Nutrients.

[51] Halfon M, Phan O, Teta D (2015). Vitamin D: A Review on Its effects on muscle strength, the risk of fall, and frailty. Biomed Res Int.

[52] Kocaer A, Sarpel T, Gökçen N, Başaran S, Coşkun Benlidayı İ (2021). Proximal muscle strength as a predictor of vitamin D insufficiency in elderly. Turk J Phys Med Rehabil.

[53] Hawkley L, Zheng B, Hedberg EC, Huisingh-Scheetz M, Waite L (2020). Cognitive limitations in older adults receiving care reduces well-being among spouse caregivers. Psychol Aging.

[54] Ransmayr G, Hermann P, Sallinger K, Benke T, Seiler S, Dal-Bianco P, Marksteiner J, Defrancesco M, Sanin G, Struhal W, Guger M, Vosko M, Hagenauer K, Lehner R, Futschik A, Schmidt R (2018). caregiving and caregiver burden in dementia home care: results from the prospective dementia registry (prodem) of the austrian alzheimer society. J Alzheimers Dis.

[55] Liu S, Li C, Shi Z, Wang X, Zhou Y, Liu S, Liu J, Yu T, Ji Y (2017). Caregiver burden and prevalence of depression, anxiety and sleep disturbances in Alzheimer's disease caregivers in China. J Clin Nurs.

[56] Davison KM, Lung Y, Lin SL, Tong H, Kobayashi KM, Fuller-Thomson E (2020). Psychological distress in older adults linked to immigrant status, dietary intake, and physical health conditions in the Canadian Longitudinal Study on Aging (CLSA). J Affect Disord.

[57] Tana C, Lauretani F, Ticinesi A, Gionti L, Nouvenne A, Prati B, Meschi T, Maggio M (2019). Impact of nutritional status on caregiver burden of elderly outpatients. A Cross-Sectional Study Nutrients.

[58] Zhao W, Zhang Y, Jia S, Ge M, Hou L, Xia X, Liu X, Yue J, Dong B (2021). The association of sleep quality and sleep duration with nutritional status in older adults: findings from the WCHAT study. Maturitas.

[59] Volkert D, Beck AM, Cederholm T, Cruz-Jentoft A, Goisser S, Hooper L, Kiesswetter E, Maggio M, Raynaud-Simon A, Sieber CC, Sobotka L, van Asselt D, Wirth R, Bischoff SC (2019). ESPEN guideline on clinical nutrition and hydration in geriatrics. Clin Nutr.

